# Hospital recruitment for a pragmatic cluster-randomized clinical trial: Lessons learned from the COMPASS study

**DOI:** 10.1186/s13063-017-2434-1

**Published:** 2018-01-26

**Authors:** Anna M. Johnson, Sara B. Jones, Pamela W. Duncan, Cheryl D. Bushnell, Sylvia W. Coleman, Laurie H. Mettam, Anna M. Kucharska-Newton, Mysha E. Sissine, Wayne D. Rosamond

**Affiliations:** 1 Department of Epidemiology, University of North Carolina Gillings School of Global Public Health, 2101 McGavran-Greenberg Hall, CB #7435, Chapel Hill, NC 27599-7435 USA; 2 Department of Neurology, Wake Forest School of Medicine, Medical Center Blvd, Winston-Salem, NC 27157 USA

**Keywords:** Hospital recruitment, Pragmatic, Cluster-randomized, Clinical trial, Stroke, Post-acute care

## Abstract

**Background:**

Pragmatic randomized clinical trials are essential to determine the effectiveness of interventions in “real-world” clinical practice. These trials frequently use a cluster-randomized methodology, with randomization at the site level. Despite policymakers’ increased interest in supporting pragmatic randomized clinical trials, no studies to date have reported on the unique recruitment challenges faced by cluster-randomized pragmatic trials. We investigated key challenges and successful strategies for hospital recruitment in the Comprehensive Post-Acute Stroke Services (COMPASS) study.

**Methods:**

The COMPASS study is designed to compare the effectiveness of the COMPASS model versus usual care in improving functional outcomes, reducing the numbers of hospital readmissions, and reducing caregiver strain for patients discharged home after stroke or transient ischemic attack. This model integrates early supported discharge planning with transitional care management, including nurse-led follow-up phone calls after 2, 30, and 60 days and an in-person clinic visit at 7–14 days involving a functional assessment and neurological examination. We present descriptive statistics of the characteristics of successfully recruited hospitals compared with all eligible hospitals, reasons for non-participation, and effective recruitment strategies.

**Results:**

We successfully recruited 41 (43%) of 95 eligible North Carolina hospitals. Leading, non-exclusive reasons for non-participation included: insufficient staff or financial resources (*n* = 33, 61%), lack of health system support (*n* = 16, 30%), and lack of support of individual decision-makers (*n* = 11, 20%). Successful recruitment strategies included: building and nurturing relationships, engaging team members and community partners with a diverse skill mix, identifying gatekeepers, finding mutually beneficial solutions, having a central institutional review board, sharing published pilot data, and integrating contracts and review board administrators.

**Conclusions:**

Although we incorporated strategies based on the best available evidence at the outset of the study, hospital recruitment required three times as much time and considerably more staff than anticipated. To reach our goal, we tailored strategies to individuals, hospitals, and health systems. Successful recruitment of a sufficient number and representative mix of hospitals requires considerable preparation, planning, and flexibility. Strategies presented here may assist future trial organizers in implementing cluster-randomized pragmatic trials.

**Trial registration:**

Clinicaltrials.gov, NCT02588664. Registered on 23 October 2015.

**Electronic supplementary material:**

The online version of this article (doi:10.1186/s13063-017-2434-1) contains supplementary material, which is available to authorized users.

## Background

Pragmatic randomized clinical trials are essential to determine the effectiveness of interventions in “real-world” clinical practice. They provide high external validity without compromising internal validity but present unique challenges in design and implementation compared with traditional randomized clinical trials [1]. To help pragmatic trial organizers measure whether they successfully incorporate pragmatism into trial design, implementation, outcome measurement, and analysis, international methodologists designed the Pragmatic Explanatory Continuum Indicator Summary (PRECIS) tool [2]. In its second iteration, PRECIS-2 included a component to measure the degree to which recruitment methods result in a study population that mirrors the real-world target patient population [2]. In their report, the authors of PRECIS-2 described pragmatic strategies for patient-level recruitment. However, in an effort to achieve direct real-world applicability, pragmatic trials frequently use a cluster-randomized methodology, with interventions that generally pose low risk to participants and can be implemented as the new standard of care. Strategies for pragmatic site-level recruitment were not included in the PRECIS-2 report, and despite policymakers’ increased interest in supporting pragmatic randomized clinical trials, to our knowledge, no studies to date have reported on the unique recruitment challenges faced by cluster-randomized pragmatic trials [3, 4].

Many randomized clinical trials—both explanatory and pragmatic—fail to recruit participants efficiently, and many trials are not undertaken because recruitment is deemed too difficult [5]. McDonald *et al.* [6] reported that more than two-thirds (69%) of trials failed to reach their target sample size within the timescale and budget originally planned. The unique design of the pragmatic cluster-randomized trial adds additional challenges and opportunities in recruitment. Here we share our experiences with recruiting hospitals to participate in the Comprehensive Post-Acute Stroke Services (COMPASS) study, with the aim of informing other investigators designing cluster-randomized pragmatic clinical trials.

## Methods

The COMPASS study is a pragmatic, cluster-randomized trial to evaluate the effectiveness of the COMPASS post-acute stroke care model versus usual care in improving self-reported functional status as well as reducing hospital readmissions, all-cause mortality, healthcare utilization, medication non-adherence, and caregiver strain. Detailed methods have been published [7]. Eligible hospitals (i.e., clusters) included all acute care hospitals in North Carolina with an emergency department that treated patients for stroke or transient ischemic attack and had the capacity to identify patients concurrent with care. Eligible patients included all patients who had been treated for stroke or transient ischemic attack and who were discharged directly home from the hospital.

To test the feasibility of our design and develop the intervention, we established a single-center “vanguard site,” Wake Forest Baptist Medical Center, a Joint Commission-certified comprehensive stroke center with an average of approximately 900 discharges of patients for stroke annually [8].

Recruitment strategies presented in this manuscript were drawn from two primary sources. Before starting recruitment, we detailed the processes and procedures that we would use in a manual of procedures. From there, we documented the evolution of the recruitment strategies employed in detailed minutes at weekly hospital recruitment coordinator-led meetings. The eight-member interdisciplinary team included a registered nurse with hospital experience, the three principal investigators, and the statistical, project, and engagement coordinators. The statistical coordinator ensured that we recruited a representative mix of hospitals, a key element in a truly pragmatic study, according to the PRECIS-2 rubric [2]. The recruitment coordinator and nurse each dedicated 0.5 full-time equivalents to recruitment for 1 year. The project coordinator relayed information from the contracts officer and the institutional review board (IRB) officer; all three of these dedicated 0.1 full-time equivalents throughout the year.

We originally proposed to recruit 50 hospitals over a 4 month period. In September 2015, we began by leveraging relationships built with the 51 hospitals already participating in the North Carolina Stroke Care Collaborative (NCSCC) [9, 10], 46 of which had provided letters of support for our proposal. The NCSCC is a prospective registry of stroke patients designed to track, measure, and improve the quality of acute stroke care. It covers 60% of North Carolina counties and was part of the Centers for Disease Control and Prevention’s Paul Coverdell National Acute Stroke Registry Program from 2002 to 2014. We then expanded recruitment to include all 110 North Carolina hospitals through webinars, teleconferences, and site visits. Between September and December 2015, we successfully contacted key personnel at all 51 NCSCC-affiliated hospitals and at 56 of the remaining 59 North Carolina hospitals.

**Fig. 1:**
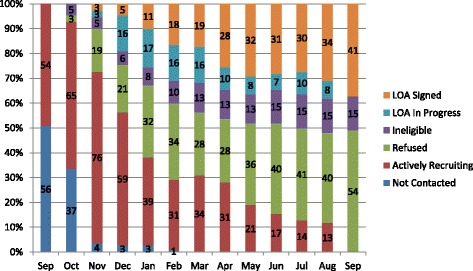
COMPASS study timeline for recruitment of 110 North Carolina acute care hospitals, September 2015 to September 2016. LOA, letter of agreement

**Fig. 2:**
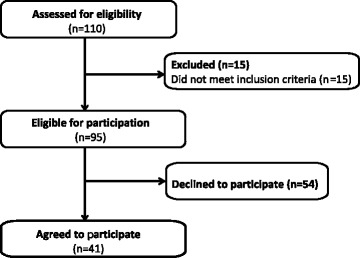
Eligibility and enrollment portion of COMPASS study CONSORT flow diagram

Thereafter (January to September 2016), we tailored our efforts to individual hospitals through individual phone calls, presentations, and site visits (Fig. 1). Through this process, 15 hospitals were deemed ineligible because they did not admit patients for stroke or transient ischemic attacks. Of the remaining 95 eligible hospitals, 54 declined and 41 (43%) ultimately chose to participate, as illustrated in the recruitment and enrollment portion of the Consolidated Standards of Reporting Trials (CONSORT) flow diagram for clinical trials in Fig. 2 [11]. The full CONSORT flow diagram and CONSORT checklist are available in Additional file 1 and Additional file 2, respectively.

At each hospital, we sought to establish a primary contact with whom we could work throughout the recruitment and enrollment process. This contact was generally at the president, vice president, or director level, although job titles varied by hospital. At sites that declined participation, we conducted a telephone interview to query the reasons for non-participation. Sites were free to report several reasons, and responses were recorded as text and categorized as patterns emerged. At successfully recruited sites, this contact was tasked with confirming that the hospital had the staffing capability to implement the intervention, given the hospital’s average annual stroke discharge volume. The intervention required a nurse-level staff member to inform the patient of the study at discharge, complete 2, 30, and 60 day follow-up calls, and complete the functional assessment during the follow-up clinic visit (7–14 days after discharge). It also specified that a nurse practitioner, physician’s assistant, or physician complete a neurological examination at the follow-up clinic visit. Oversight by an on-site principal investigator was also required. All staff serving in these roles were required to complete training on human subjects research and information privacy and security through the Collaborative Institutional Training Institute (CITI) [12] (or equivalent) and maintain certification per institutional guidelines. These roles were specified in detailed job descriptions and the letter of agreement. Additional details have been published [7].

In an effort to mimic a “real-world” environment, financial incentives for participation are limited and were not designed to cover the full cost of staff time [2]. All participating hospitals receive limited per-case direct reimbursement for ascertaining and enrolling eligible cases. Hospitals in the intervention arm receive additional limited per-case reimbursement for completing the follow-up visit. The 7–14 day clinic visit was designed in such a way as to meet the billing requirements for Centers for Medicare and Medicaid Services Transitional Care Management (TCM) and Chronic Care Management (CCM).

As a pragmatic, patient-centered trial, COMPASS incorporated patient perspectives throughout the study design, including an iterative consenting design process, whereby stroke survivors and caregivers worked with the vanguard hospital’s IRB office to develop a consenting protocol that maximized participation and provided a strongly patient-centered approach. As the intervention changes hospitals’ structure and process of care for all patients having had stroke or transient ischemic attack who are discharged home, patients cannot fully opt out without seeking care elsewhere; however, we designed a consenting process that provided several opportunities for education and consenting, with key points at discharge and prior to the 90-day telephone survey. Interviewers for the 90-day survey were blinded to hospitals’ randomization assignment. Additional details of this innovative consent design and methodology have been published [7].

Informed by experience and reported benefits of central IRBs for multicenter trials [13–15], we offered sites the option to participate in the COMPASS central IRB to avoid lengthy start-up delays caused by repeated IRB reviews of the same protocol and to aid recruitment of small hospitals without IRBs.

Once the letter of agreement was signed, IRB approval was received, and CITI training was complete for all on-site COMPASS staff, hospitals were randomized in pairs, each matched with another hospital of similar annual stroke discharge volume (<100, 100–299, 300+ patients) and primary or comprehensive stroke center certification status (“yes” or “no” as designated by the Joint Commission, an independent, voluntary national hospital accreditation and certification organization). Using a stepped-wedge design, hospitals were randomized to either receive the COMPASS intervention at the beginning of the study or after a period of providing their usual care, to provide a control group [7].

A COMPASS statistician ensured enrollment of a sufficient number of hospitals in each of the six strata for stroke discharge volume and stroke center status, using the average estimated number of patients who had stroke or transient ischemic attack who were discharged home each year from each hospital in sample size calculations. These estimates were confirmed with hospital staff. Additional details have been published [7].

## Results

The 41 hospitals successfully recruited to participate in the COMPASS study are distributed across North Carolina, with the majority located in the central “Piedmont” region, where much of the state’s population resides (Table 1). Compared with all eligible hospitals in North Carolina, participating hospitals were more likely to be certified as primary or comprehensive stroke centers (59% vs. 41%) and to have higher annual stroke discharge volumes (29% vs. 17%). Participating hospitals were less likely to be in rural or small-town areas (10% vs. 23%) but included a similar proportion of critical access hospitals, compared with all eligible hospitals in North Carolina.

**Table 1:** Selected characteristics of COMPASS hospitals, compared with North Carolina acute care hospitals, 2016

	All North Carolina hospitals *N* = 110	Eligible North Carolina hospitals *N* = 95	Participating hospitals *N* = 41
Primary or comprehensive stroke center, ^a^ *n* (%)	39 (35%)	39 (41%)	24 (59%)
North Carolina Stroke Care Collaborative participant, *n* (%)			
ᅟActive	48 (44%)	47 (49%)	21 (51%)
ᅟIntermittent or inactive	26 (24%)	25 (26%)	11 (27%)
ᅟNever	36 (33%)	23 (24%)	9 (22%)
Critical Access Hospital, *n* (%)	21 (19%)	15 (16%)	5 (12%)
Geographic region, *n* (%)			
ᅟCentral Piedmont	48 (44%)	43 (45%)	18 (44%)
ᅟWestern	23 (21%)	21 (22%)	11 (27%)
ᅟEastern	39 (35%)	31 (33%)	12 (29%)
Medical school affiliation, ^b^ *n* (%)			
ᅟMajor	7 (6%)	6 (6%)	3 (7%)
ᅟMinor	12 (10%)	12 (12%)	5 (12%)
ᅟNone	91 (83%)	77 (81%)	33 (80%)
2013 stroke discharge rate, *n* (%)			
ᅟ < 100	53 (48%)	39 (41%)	11 (27%)
ᅟ100–299	41 (37%)	40 (42%)	18 (44%)
ᅟ300+	16 (15%)	16 (17%)	12 (29%)
Hospital bed size, *n* (%)			
ᅟ < 100	39 (35%)	29 (31%)	15 (37%)
ᅟ100–299	48 (44%)	44 (46%)	16 (39%)
ᅟ ≥ 300	23 (21%)	22 (23%)	10 (24%)
Urban-rural classification, *n* (%)			
ᅟRural or small town	28 (25%)	25 (23%)	4 (10%)
ᅟMicropolitan	30 (27%)	27 (28%)	15 (37%)
ᅟMetropolitan	52 (47%)	43 (45%)	22 (54%)
Ownership, *n* (%)			
ᅟPrivate, not-for-profit	53 (52%)	47 (51%)	18 (46%)
ᅟPrivate, for-profit	11 (11%)	10 (11%)	3 (8%)
ᅟLocal	15 (15%)	14 (15%)	6 (15%)
ᅟHospital district or authority	15 (15%)	13 (14%)	8 (21%)
ᅟOther	8 (8%)	8 (9%)	4 (10%)
ᅟUnknown or missing	8	3	2

^a^Certified by the Joint Commission, an independent, national, voluntary hospital accreditation and certification organization

^b^Centers for Medicare and Medicaid Services categories; “Minor” includes graduate or limited medical school affiliation or participation

The majority (*n* = 36, 88%) of hospitals utilized the COMPASS central IRB option; only five hospitals (12%), three of which were in the same health system, opted to participate through internal IRBs.

**Fig. 3:**
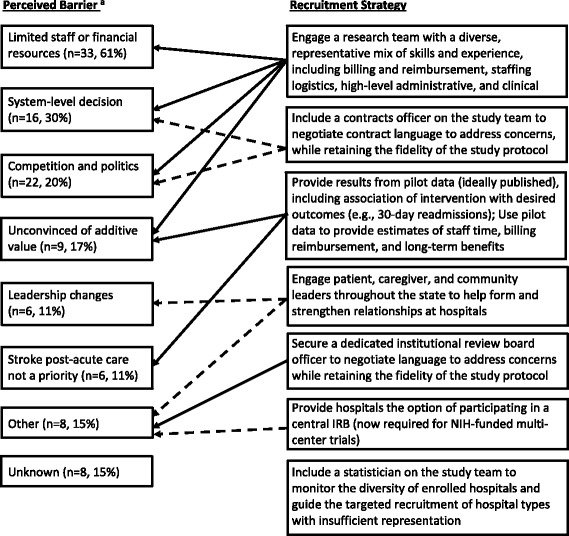
Perceived barriers to COMPASS study participation and successful recruitment strategies, September 2015 to September 2016. IRB, institutional review board; NIH, National Institutes of Health. ^a^ Perceived barriers are not mutually exclusive; therefore, percentages sum to >100%

Over half (61%) of the 54 hospitals that declined participation cited insufficient staff or financial resources to meet the staffing requirements to implement the intervention (Fig. 3). Nearly one-third did not participate as a result of a decision at the hospital system level, and one-fifth cited the inability to organize support of hospital or system-level decision-makers. Other reasons included doubts regarding the additive value of participation, administrative leadership changes, low priority placed on improving post-acute stroke care, concerns about impact on relationships with local primary care physicians, and informed consent, sustainability, or manuscript authorship control. We did not receive responses to our request for interviews at eight hospitals and were unable ascertain their reasons for non-participation. Key strategies employed to address perceived barriers to participation are listed briefly in Fig. 3 and are described in greater detail next.

## Discussion

Although we recruited a sufficient number of hospitals to meet statistical power requirements for the primary outcome measures of the trial, the process took longer and was more complex than anticipated. Ultimately, we successfully recruited 43% of the 95 eligible hospitals and developed strategies that should prove valuable for other researchers planning cluster-randomized pragmatic trials.

Although the current literature on hospital recruitment is sparse, we developed initial strategies based on the available evidence and our experiences in recruitment for the NCSCC. Whicher *et al.* [16] and Anderson *et al.* [17] discuss the importance of identifying and engaging gatekeepers, particularly in recruiting large health systems, where there may be many gatekeepers and varying types of governance. Informed by such research, before initiating formal recruitment, we sought contact information for as many key clinical and administrative leaders as we could at all 110 acute care hospitals in North Carolina. Helpful in identifying these personnel were NCSCC hospital contacts, local stroke organizations (North Carolina Stroke Association, North Carolina Stroke Advisory Council and the Area Agency on Aging), and other community partners and stakeholders, including faith-based and counseling networks. Additional details of community and stakeholder engagement throughout the COMPASS design have been published [18].

Patsopoulos [3] emphasized the importance of achieving a heterogeneous, representative mix of participants and settings, which is also important for recruitment of pragmatic trials, noting the challenge this can pose in terms of sample size. Throughout the recruitment process, to ensure that we achieved a pragmatic hospital sample with sufficient representation from each of the six strata for stroke discharge volume and stroke center status, power calculations helped drive the targeted recruitment of hospitals in strata that had not yet met their target sample sizes.

A common recruitment challenge in any cluster-randomized trial is that of patient consent [1, 19]. Cluster-randomized trials frequently utilize a waiver of consent for the intervention, with consent obtained only for the collection of follow-up data, in an effort to ensure a truly representative patient population. While this simplifies patient-level recruitment, it can add challenges at the cluster level [1]. Further, our consent model incorporated stroke survivor and caregiver perspectives, which created processes that were somewhat different from traditional clinical trial procedures, and which needed to be discussed with administrators at recruited hospitals. In anticipation of these challenges, we secured a dedicated member of the central IRB, who was available to work directly with local IRBs and administrators and answer questions; thus lending confidence in the process.

To accelerate recruitment at the cluster level, many multicenter trials also provide participating sites with the option of participating in a central IRB; this is now a requirement for multicenter trials funded by the National Institutes of Health [20]. Providing a central IRB option was critical to meeting our goal of recruiting a pragmatic, representative mix of hospitals, particularly at smaller sites that do not traditionally participate in clinical research or that do not have local IRBs. Also, our central IRB coordinator reached out directly to primary contacts at each site to establish whether the hospital had participated in research in the past, whether it had a local IRB, and whether that local IRB was willing to join the central COMPASS IRB, and worked through related concerns or challenges.

The Agency for Healthcare Research and Quality [21] reported that hospitals are most likely to participate in a quality improvement registry because of interest in the research question or because it helps them fulfill reimbursement or regulatory requirements. This report also cited staff time and costs for data collection as a key barrier to participation. The Institute of Medicine [22] underscored the importance of tailoring recruitment strategies and messages based on stakeholder perspectives. Understanding that hospital administrative leadership must guard financial resources and juggle competing priorities, and that clinical personnel focus more on additional staff time resources, we tailored recruitment materials to different decision-makers. To appeal to clinical staff, our materials provided staff time estimates from pilot data at the COMPASS vanguard site. For administrators, we emphasized potential benefits, such as cost savings through bundled payments and improved reimbursement for the follow-up visit.

Despite discussing these and other benefits of participation with hospitals, by December 2015, we had received only five letters of agreement. Like Rosen *et al.* [23], we found that hospitals were more likely to participate in the study if they had an “entrepreneurial culture” already in place (i.e., one that promotes taking risks, innovation, and quality improvement); all five of the first hospitals that signed COMPASS letters of agreement in the first 4 months were hospitals that actively engaged administrators and physician and nurse leadership at the outset of discussions. These five hospitals, as well as 16 others indicating strong interest, also had in common prior experience of participating in clinical research and a clear “champion” of the project with decision-making power.

During the first 4 months of recruitment, we also had 21 refusals. We reviewed the reasons for these refusals and assessed our inability to make a successful connection at 39 hospitals. We developed the following strategies to address these issues and improved our remaining recruitment efforts.

### Limited resources

Consistent with the current literature, the primary reason that hospitals declined to participate was their concern of insufficient staff or financial resources to carry out the intervention (61%). Perceived resource concerns varied by hospital. For example, at some sites the primary concern was whether they had a staff member to complete case identification and enrollment; at others, patient enrollment was feasible, but staff could not identify a provider to complete the post-acute follow-up visit. For others, the modest financial compensation we provided was deemed insufficient to enable participation.

To overcome resource challenges, we leveraged the study’s pragmatic design to provide flexible approaches where needed to ensure that the intervention could be faithfully implemented while making allowances for different staffing levels, financial resources, organizational structures, cultures, and inter-institutional relationships. For example, at one hospital that had difficulty identifying staff to ascertain and enroll cases, we identified a local paramedicine practice to serve in this role. A key element in true pragmatism is how consistent an intervention is with the “usual” organization and delivery of care provided [2]; being able to provide this kind of flexibility to hospitals enhanced their ability and willingness to participate.

### System-level decisions, competition, and politics

In the first 4 months of recruitment, two hospital systems (eight hospitals) declined participation owing to market competition and complex institutional relationships with the COMPASS vanguard hospital. Soon after, it became clear that two more hospital systems were leaning toward refusal for similar reasons. To avoid refusal by these systems—both of which were large, research-academic health systems—we engaged in high-level conversations with hospital system administration. Recruitment of a research team and partners with a diverse, representative mix of skills and experience is a hallmark of a truly pragmatic trial [1], and without the support of engaged partners throughout the state (in this case, hospital administrative leadership with an understanding of the intense and complex competition for research and innovative interventions), these successful conversations would not have been possible.

Also, early on in the recruitment process, we engaged a member of the COMPASS vanguard site’s contracts department to work directly with hospitals’ contracts officers to agree on contract language to ensure that concerns regarding the formal relationships of participating institutions were being addressed, while retaining the fidelity of the COMPASS protocol.

Other types of competitive forces also posed challenges. At three hospitals that declined participation, as well as at a number of others that did ultimately participate, the concern was raised that participation in the COMPASS study might strain already delicate relationships with community primary care physicians. These sites were concerned that local primary care physicians unaffiliated with the hospital might view the hospital’s use of a single practitioner for the follow-up visit as anti-competitive or an attempt by the hospital to “steal patients.” To address this concern, we engaged clinical experts (who explained how the COMPASS intervention actually supports primary care physicians and encourages follow-up visits), billing experts (who shared details on billing codes still available to primary care physicians even if the TCM or CCM codes were used by the COMPASS practitioner), as well as a COMPASS family medicine physician (who discussed this issue with stakeholders from a primary care physician’s perspective). We also addressed this concern by working together with stakeholders to develop pragmatic, creative solutions that still upheld the integrity of the intervention. For example, at one hospital, we created two sites for the post-discharge follow-up visit: one at the major local primary care clinic for patients whose primary care physicians were part of this network, and another in a hospital-based clinic for all other patients. Having a dedicated contracts officer to negotiate contract language to provide agreements that met these more complex, individualized arrangements, while retaining the fidelity of the COMPASS protocol, was instrumental.

### Unconvinced of additive value

Nine hospitals (17%) declined participation because they did not believe the COMPASS intervention would provide sufficient value beyond their usual care practices. At some sites this appeared to be due to a lack of understanding of the clinical value of the intervention and the science behind it, while at others there appeared to be a lack of understanding or confidence in the financial value of the intervention, particularly given the limited financial incentives provided by pragmatic trials such as ours [2]. To address this, we matched the skills and expertise of COMPASS team members with those of hospital decision-makers. For example, in sites where neurology practices were considered for the post-acute follow-up site, the COMPASS neurologist shared first-hand experience as to how this could work in practice. Likewise, COMPASS billing specialists and clinicians from the vanguard site responded to concerns about long-term financial value and sustainability. A particularly valuable resource was the published pilot data from our vanguard site, indicating that the COMPASS care model was associated with a 48% reduction in 30 day hospital readmissions [8].

Essential in this process was determining the perceived drivers and barriers for each stakeholder, finding the point of intersection of our interests, and creating pragmatic, individualized strategies. For example, through discussions with administrators at one community access hospital, we found that our partnership could help the hospital improve its visibility and position in the community by demonstrating its commitment to providing innovative, high-quality stroke care.

### Other barriers

Hospitals that signed letters of agreement early in recruitment were those with which we had established a strong working relationship through the NCSCC and engaged a clear “champion” (i.e., a decision-maker with a dedication to improving stroke care, willingness to consider a new approach, and institutional clout). At other hospitals, however, identifying and establishing relationships with gatekeepers was less straightforward. While some hospitals took a collaborative approach to decision-making, involving various clinical and administrative leaders, others employed a single decision-maker. At nine eligible hospitals, our failure to cultivate buy-in from a single gatekeeper resulted in refusal to participate. Also, while decision-making at some hospitals was controlled at the hospital system level, at others, individual hospitals were free to make their own decisions about participation.

At hospitals where we had difficulty identifying or establishing relationships with gatekeepers, we had success with “relational recruiting” by leveraging community partnerships. With its patient-centered focus, COMPASS has strong relationships with community stakeholders throughout the state, who were actively involved in the design and implementation of COMPASS and were a critical resource for helping us form relationships at hospitals where we did not previously have a relationship. Similarly, in a volatile market for hospital ownership and management, leadership changed at a number of hospitals during the recruitment period, and these community partners were also helpful in establishing relationships with new leadership and confirming community buy-in for our study.

While our aim is to provide strategies that will be helpful to a broad array of trial organizers designing pragmatic cluster-randomized trials, our findings are limited to the experiences of one trial and may not be generalizable to trials with different study populations, provider types, or intervention requirements.

## Conclusions

Although our recruitment process took three times as long as anticipated and considerably more staff resources, we successfully recruited 43% of eligible North Carolina hospitals and enrolled a sufficient number of cluster units to meet our design requirements. Through this process, we learned valuable lessons in how to recruit hospitals for a multicenter pragmatic randomized clinical trial.

Building and nurturing relationships was paramount throughout, from identifying stakeholders and gatekeepers, building a team with a diverse and representative skill mix, and engaging partners and team members in discussions with decision-makers. Essential in this process was the importance of listening, focusing on the stakeholder perspective, building trust, and seeking mutually beneficial solutions. Further, providing a central IRB option, having IRB and contracts team members, and sharing results and experiences from pilot work were valuable recruitment strategies. Successful site-level recruitment requires significant preparation, planning, and flexibility. We hope that the strategies we developed as our recruitment efforts evolved will assist future trial organizers in designing strong cluster-randomized pragmatic trial recruitment methodologies.

## Additional files

Additional file 1: COMPASS study CONSORT flow diagram (DOCX 52 kb)

Additional file 2: CONSORT 2010 randomized trial reporting checklist: the COMPASS study (DOC 216 kb)

## Abbreviations

CCM: Chronic Care Management
CITI: Collaborative Institutional Training Institute
COMPASS: Comprehensive Post-Acute Stroke Services
CONSORT: Consolidated Standards of Reporting Trials
IRB: Institutional review board
NCSCC: North Carolina Stroke Care Collaborative
PRECIS: Pragmatic Explanatory Continuum Indicator Summary
TCM: Transitional Care Management
