# Supplementary material for: Hospital recruitment for a pragmatic cluster-randomized clinical trial: Lessons learned from the COMPASS study
Source: Trials. 2018 Jan 26;19:74. doi: 10.1186/s13063-017-2434-1 (PMC5787294; doi:10.1186/s13063-017-2434-1)
Supplement: Supplementary file 1 — COMPASS study CONSORT flow diagram (DOCX 52 kb) [file 13063_2017_2434_MOESM1_ESM.docx]

*Additional file 1: Figure S1.* COMPASS study CONSORT flow diagram

**Eligible for participation**

(n=95)

**Agreed to Participate**

(n=41)

**Declined to participate (n=54)**

**Excluded (n=15)**

Did not meet inclusion criteria (N=15)

**Assessed for eligibility**

(n=110)

**Two paired as single randomization unit**

**Randomized**

(n=40)

**Intervention**

(n=20)

**Usual Care**

(n=20)
